# Characterization of the Low Molar Ratio Urea–Formaldehyde Resin with ^13^C NMR and ESI–MS: Negative Effects of the Post-Added Urea on the Urea–Formaldehyde Polymers

**DOI:** 10.3390/polym10060602

**Published:** 2018-05-31

**Authors:** Hui Wang, Ming Cao, Taohong Li, Long Yang, Zhigang Duan, Xiaojian Zhou, Guanben Du

**Affiliations:** 1 The Yunnan Province Key Lab of Wood Adhesives and Glued Products, Southwest Forestry University, Kunming 650224, China; w20030608016@126.com (H.W.); caominghappy@swfu.edu.cn (M.C.); long133109070@126.com (L.Y.); emailofdzg@126.com (Z.D.); xiaojianzhou@hotmail.com (X.Z.); 2 Key Lab for Forest Resources Conservation and Utilisation in the Southwest Mountains of China, Southwest Forestry University, Ministry of Education, Kunming 650224, China

**Keywords:** urea–formaldehyde resin, post-added urea, mole ratio, debranching effect

## Abstract

The structural changes during three-step synthesis of low-molar ratio urea–formaldehyde (UF) resin were tracked by quantitative ^13^C nuclear magnetic resonance (^13^C NMR) analysis and electrospray ionization-mass spectrometry (ESI–MS). Condensations that produced polymers were found to be linked by ether bonds in addition to hydroxymethylation reactions at the first alkaline stage with a formaldehyde to urea ratio of 2:1. Considerable formation of branched methylene linkages, with the highest content among all the condensed structures, was the key feature of the acidic stage. Notable changes were observed for the chemical structures and molecular masses of the resin components after the formaldehyde to urea molar ratio was lowered to 1.2 by adding post-urea at the final alkaline stage. Specifically, most of the branched hydroxymethyl groups on the polymers were cleaved, resulting in a significant decrease in the branching degree of the polymers. The performance degradation of the UF resin was attributed to this debranching effect and the production of components with low molecular masses. Based on the observations, the curing pattern of low molar ratio UF resin was postulated and branched polymeric formaldehyde catcher bearing urea-reactivity was proposed.

## 1. Introduction

Urea–formaldehyde (UF) resin has been the most important wood adhesive resin during the past century due to its good performance and low manufacturing cost. However, formaldehyde emissions from the wood-based panel products has been challenging its applications. To address the formaldehyde emission problem, the molar ratio of formaldehyde to urea has been lowered to 0.9–1.2 [1,2]. As a result, formaldehyde emission levels have been significantly lowered, but as a result, the performance of the resin has been largely sacrificed, especially the water resistance performance.

Currently, three-step synthesis is the most widely used procedure for UF resin synthesis [1,2]. In the first step, urea-formaldehyde reactions are performed under alkaline conditions at a molar ratio of F/U = 1.8–2.5. In this stage, hydroxymethylation is believed to be the dominant reaction that produces mono-, di-, and tri-hydroxymethylureas. In the second step, the pH is adjusted to acidic ranges, generally from 4.0 to 5.5, for the condensation between hydroxymethylureas. In this stage, polymers containing methylene linkages (–NR–CH_2_–NR–) and methylene ether linkages (–NR–CH_2_–O–CH_2_–NR) are produced, as well as a portion of cyclic species known as urons. The F/U molar ratio is generally maintained above 1.8 at this stage to avoid precipitation. After the acidic condensation, the pH is adjusted back to alkaline and the additional urea (the second urea) is added to lower the F/U molar ratio to 0.9–1.2, aiming to capture the un-reacted formaldehyde. Although intensive studies have been completed to investigate the chemical structures of resin polymers by employing nuclear magnetic resonance d(NMR) analysis [3,4,5,6,7,8,9,10,11,12,13,14,15], our survey of the literature showed that the effects of post-added urea on the UF polymers were not focused or well-discussed. However, this issue is important for better understanding the relationship between low molar ratio and poor performance of the resin. In this study, quantitative ^13^C NMR determinations were performed to track the typical three-step synthesis of UF resin, aiming to determine the changes in the chemical structures occurring after additional urea was added.

In addition to the chemical structure, the molecular mass distribution of a polymer is also an important characteristic related to the performance of the resin. The light scattering and gel-permeation chromatograph (GPC) were used to determine the molecular mass distribution of UF resin in the 1990s [1]. However, only weight-averaged molar masses of the polymers can be obtained using these techniques; the accurate mass of a molecule related to the structure of a polymer cannot be provided. Some studies have shown that the laser desorption/ionization time-of-flight mass spectrometry (MALDI-TOF MS) was suitable for studying the molecular masses of wood adhesive resins [16,17]. By using the “soft ionization” technique, MALDI-TOF MS mainly produces molecular ions and few fragments, facilitating the analysis of the spectrum. Another mass spectrometry technique, the electrospray ionization mass spectrometry (ESI-MS) also uses the soft ionization technique. It has been widely used in the analysis of peptides and proteins, but its application in analysis of wood adhesive resins is rather limited. In this study, this technique was introduced to track the changes in the molecular mass distribution of UF resin components during synthesis and to investigate the influences of the urea added at the final alkaline stage. By combining the results of ^13^C NMR and ESI–MS, the mechanism of the low F/U ratio that results in the poor performance of UF resin can be better understood.

## 2. Experimental

### 2.1. UF Resin Sample Preparation

The analytical reagent grade (AR) urea, sodium hydroxide, formic acid, and formaldehyde (37%, *w*/*w*) containing about 7% methanol were bought from Sinopharm Chemical Reagent Co., Ltd., (Shanghai, China). Formaldehyde solution was placed in the reactor and the pH was brought to 8.5–9.0 using 20% NaOH and then heated to 50 °C. Then, the first urea was mixed under mechanical stirring to obtain a F/U molar ratio of 2:1. After the urea dissolved completely, the pH of the mixture was re-adjusted to 8.5–9.0. The reaction was maintained at 90 °C for 40 min and then the pH of the mixture was lowered to 4.5–5.0. When the cloudy point was observed, the pH was brought back to 8.0–8.5 and the mixture was cooled to 60 °C. The second urea was added to the mixture to obtain the desired F/U molar ratio of 1.2. The reaction was maintained at 60 °C for 20 min and then the mixture was quickly cooled to room temperature.

Sampling procedure: (1) At the end of first alkaline stage: UF1. (2) During the acidic stage: UF2 (10 min), UF3 (30 min), and UF4 (end point). (3) At the end of second alkaline stage: UF5.

### 2.2. Procedure for Quantitative ^13^C-NMR Determination

The ^13^C nuclear magnetic resonance (^13^C NMR) spectra were measured using a Bruker AVANCE 600 spectrometer (Bruker Corporation, Billerica, MA, USA).

For each sample, a 400 μL liquid UF sample was directly mixed with 100 μL of dimethyl sulfoxide (DMSO-_d6_) for ^13^C NMR determination. The spectra were recorded with a pulse angle of 90 degrees (12 μs). A 6-s relaxation delay was used to secure quantitative results of methylenic carbons, which had *T1* values of 0.16 s or smaller, measured by the inversion recovery method [4]. To achieve a sufficient signal-to-noise ratio, the inverse-gated proton decoupling method was used. The spectra were recorded at 150 MHz with 400–600 scans accumulated. The observed chemical shifts were assigned by referring to the assignments in the literature [3,4,5,6,7,8,9,10,11,12,13,14,15].

Except for the methanol peaks at 50 ppm and methoxyl ether at 56 ppm, the peaks that were smaller than 100 ppm from methylene carbons were integrated and summed. The relative contents (%) (or molar distribution) of all methylene carbons were calculated as the ratio of the integral value of each type of methylene carbon over the total value of all methylene carbons. The relative contents of different carbonyl carbon atoms in urea were also calculated using the same method.

### 2.3. Procedure for ESI-MS Spectrometry

Mass spectrometric studies were performed on a quadrupole time-of-flight (Q-TOF) high-resolution mass spectrometer (Q-TOF LC/MS 6540series, Agilent Technologies, Santa Clara, CA, USA) coupled with electrospray ionization (ESI). The data were acquired using Mass Hunter Workstation software. The detection was performed in positive ESI mode. The MS parameters were optimized as follows: the fragmentor voltage was set at 135 V, the capillary was 3500 V, the skimmer was set to 65 V, and nitrogen was used as the drying (350 °C, 8 L/min) and nebulizing (30 psi) gas. The molecular ions produced were M + H^+^, M + Na^+^, and M + K^+^. In some cases, the complex ions, 2M^+^ and 3M^+^, in which two or three molecules were associated with H^+^, Na^+^, and K^+^ were observed. For the ESI-MS sample preparation, 1 μL liquid UF resin was dissolved in 1 mL methanol.

## 3. Results and Discussion

### 3.1. ^13^C NMR Results

**Figure 1:**
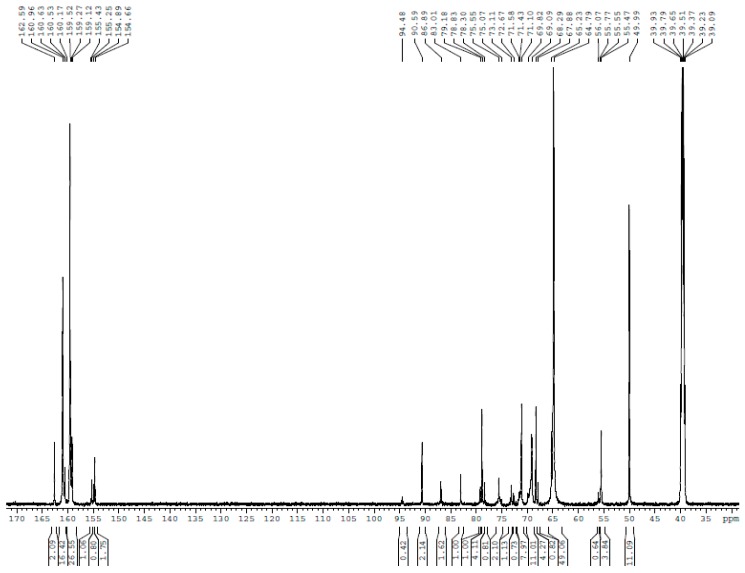
The ^13^C nuclear magnetic resonance (^13^C NMR) spectrum of sample UF-1.

**Figure 2:**
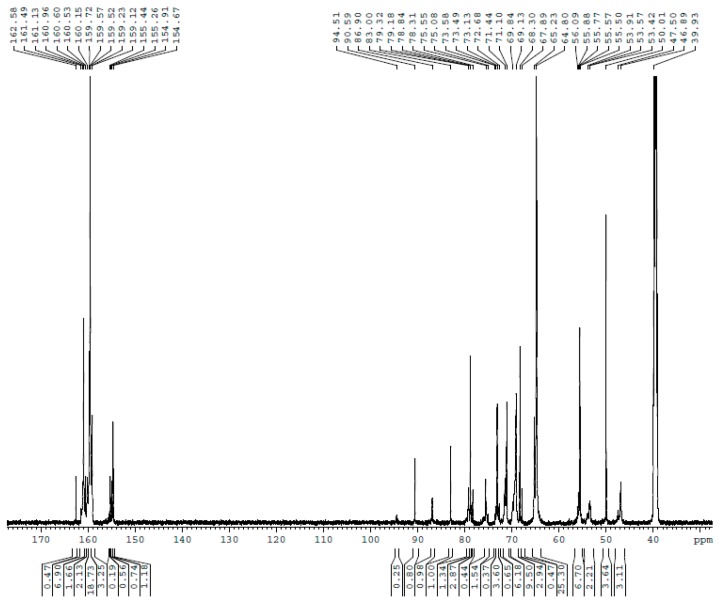
The ^13^C NMR spectrum of sample UF-2.

**Figure 3:**
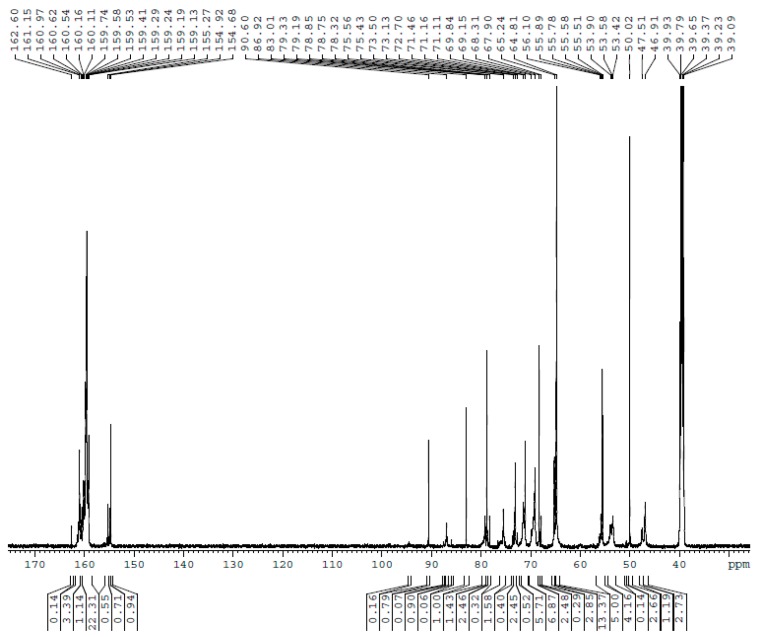
The ^13^C NMR spectrum of sample UF-3.

**Figure 4:**
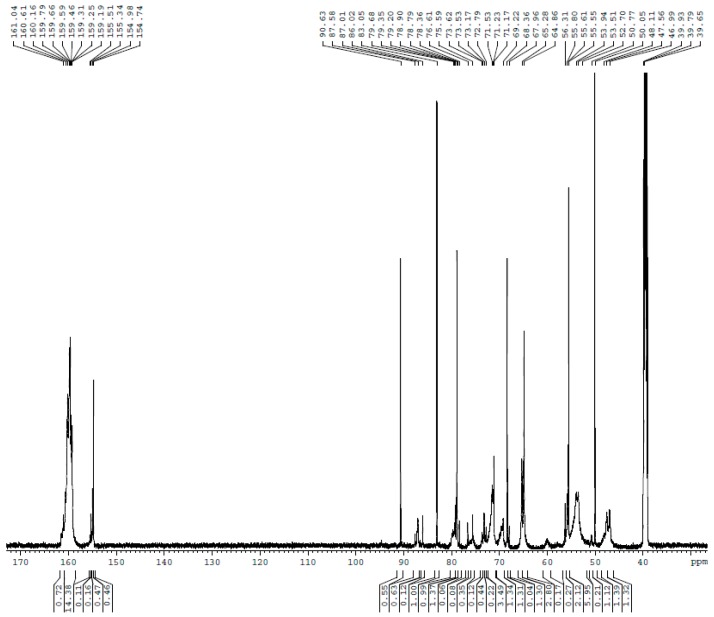
The ^13^C NMR spectrum of sample UF-4.

**Figure 5:**
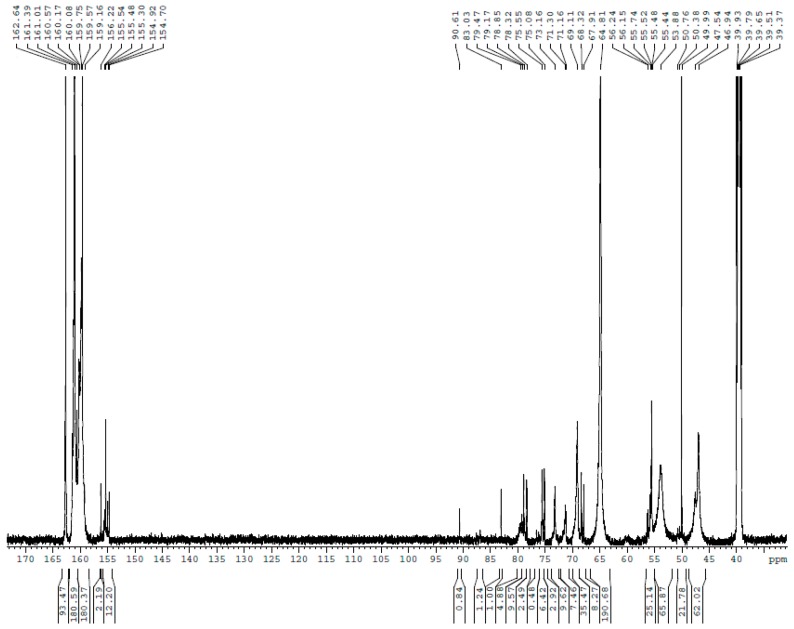
The ^13^C NMR spectrum of sample UF-5.

The ^13^C NMR spectra for UF1–UF5 are shown in Figure 1, Figure 2, Figure 3, Figure 4 and Figure 5, respectively. The assignments and calculated relative contents for different type of carbons are displayed in Table 1.

The classical theory of UF resin highlights that the urea-formaldehyde reactions under alkaline conditions dominantly produce hydroxymethylureas. However, the quantitative ^13^C NMR results for UF-1 in Table 1 show that 27% formaldehyde was converted to different methylene ether carbons. Specifically, the linear ether linkage at 68–69 ppm was dominant, accounting for 18%, and the uron species formed through hydroxymethylurea self-condensations at 155–158 ppm accounting for 7%. These results indicate that considerable condensation occurred at this stage. Therefore, describing the UF reaction under alkaline conditions with the simple word “hydroxymethylation” would not be accurate.

Methylene linkages were not formed in UF1. This is consistent with the classical theory. However, different types of methylene linkages were shown to efficient form and become dominant at the later stage of reactions if the reactions were accelerated under strong alkaline conditions (pH > 12) in our study [18]. Moreover, the rearrangement of methylene ether linkages to methylene linkages was also observed. These results can be attributed to the fact that methylene linkages are more thermodynamically stable than ether linkages. This is similar to the situation under acidic conditions. A large problem for the synthesis of UF resin under strong alkaline conditions is the strong Cannizzaro reaction of formaldehyde that produces a large amount of methanol [18].

Containing stable methylene linkages as the main condensed structures is accepted as a primary requirement for a UF resin with normal bonding strength. However, this concept is somewhat ambiguous. The structural requirement should not be restricted to a certain type of chemical bond. Instead, whether the polymers have a highly branched structure that assures further condensation and a sufficiently cross-linked network during the curing process should be the key factors. Under relatively weak alkaline conditions, and within a limited timeframe, UF reactions mainly produce polymers with a linear ether structure. This is the reason that the UF resin cannot be synthesized under alkaline conditions. As for the molecular mass, following ESI–MS results show that the molecular masses of the UF polymers formed at the first stage were not significantly smaller than those of the final resin.

As can be seen in UF2, once the reactions occurred at acidic pH, linear methylene linkages at 46–47 ppm and branched linkages at 53–54 ppm began to form. Ether linkages still increased. When the reactions came to UF3 (30 min), more methylene linkages were formed, but ether linkages began to decrease.

UF4 corresponds to the end point of the acidic stage. Significant changes can be seen here. The content of linear ether linkages (68–69 ppm) decreased by 50% in contrast with the highest value. The methylene linkages became dominant; the branched structure at 54 ppm accounted for 23%, representing the highest content of all the condensed structures.

Note that the content of uron species was maintained the same level of 7% during the entire process. This is different from the observation in an earlier study [19] where the uron content fluctuated with the change in pH. Our previous theoretical calculations using the quantum chemistry method suggested that the uron structure should be thermodynamically as stable as chain methylene linkages [20]. However, a higher kinetic energy barrier and the requirement for 1,3-dihydroxymethylureas or trihydroxymethylureas results in their relatively lower content than methylene linkages. Direct evidence is provided by the content of uron methylene carbons that were observed to be above 20% when the F/U molar ratio was above 2.5 or higher [14,18,19]. The urons were efficiently formed at the early stage of the reaction under strong alkaline or acidic conditions.

From UF3 to UF4, the content of free formaldehyde increased from 5 to 9% with the conversion of chain ether linkages to methylene linkages. During the hot press process in manufacturing wood-based panels, and curing (further condensation and re-arrangement) releases more formaldehyde. This is why additional urea must be added after the acidic stage. To capture the formaldehyde released by the breakage of the condensed structure in the resin during use of wood-based panel products, excessive urea becomes necessary. However, use of additional urea may cause negative influences on the UF polymer structure.

**Figure 6:**
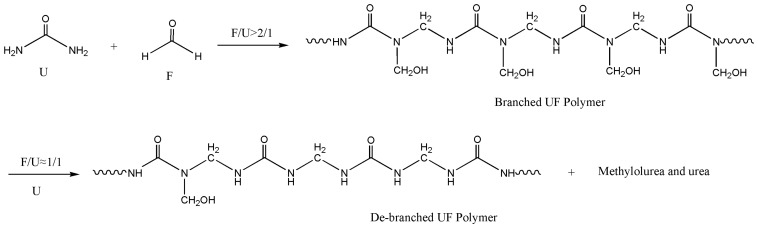
Debranching effect of the post-added urea.

After the addition of the second urea, several obvious changes occurred in the polymer structure. The most significant change was the content of the hydroxymethyl groups. Specifically, the type I mono-substituted hydroxymethyl group at 65–66 ppm increased from 16% in UF4 to 46% in UF5. Reactions between additional urea and free formaldehyde that produced considerable mono-hydroxymethylurea contributed about 9% to this change as the free formaldehyde decreased from 9.19% to 0.75%, indicating that the second urea had eliminated most of the un-reacted formaldehyde. This is the benefit of additional urea. However, the content of type II hydroxymethyl group at 70–71 ppm decreased from 13.94% to 1.82%. This type of hydroxymethyl group corresponds to N,N-di hydroxymethylurea at the end of a polymer as –N(CH_2_OH)_2_, or in the internal part of a condensed chain as –N(–CH_2_–)–CH_2_OH. The decrease of this group means a decrease in the branching degree of the polymers. A portion of the branched UF polymers was converted to linear polymers. Corresponding changes were also seen from the significant decrease in type II branched methylene linkage at 54–55 ppm and the significant increase in the type I linear linkages at 46–47 ppm. This debranching effect made the polymers more linear as demonstrated by the reaction shown in Figure 6. The post-added urea caused the movement of the hydroxymethyl group from polymers to urea.

From a chemical point of view, the loss of bonding strength of the UF resin under humid conditions can be mainly attributed to the instability of the chemical structures toward hydrolytic attack. A higher F/U molar ratio generally means a higher branching degree of resin polymers. Highly branched polymers ensure a compact cured network that prevents fast water permeation, so failure of a resin can be definitely delayed. As the F/U molar ratio decreases, the cured-network becomes fragmentary or incomplete. This is an easy concept. However, some studies found that the hydrolytic stability of UF resin increased with the decrease in F/U molar ratio [21,22]. The crystalline region detected for cured UF resin with a F/U molar ratio lower than 1.2 was supposed to be responsible for the better water resistance. Note that these studies only investigated the resin cured alone, not that cured in contact with wood. An earlier study pointed out that the UF resin cured in the presence of wood can only be amorphous [23]. Interestingly, a more detailed study using field emission scanning electron microscopy (FE-SEM), Fourier transform infrared (FTIR) spectroscopy, and wide-angle X-ray diffraction (WAXD) clearly demonstrated the crystallization of cured low molar ratio UF resin in contact with wood, but the authors implied that the presence of crystallinity may negatively impact the cohesive strength of the resin [24]. Another study on the hydrolytic stability of solid UF resin (not cured) also suggested that high molar ratio UF resin showed a higher susceptibility to hydrolysis than low molar ratio resin [10]. Understanding the higher susceptibility of highly branched resin to hydrolysis is not difficult. A UF resin with a high molar ratio contains more un-reacted (un-condensed) hydroxymethyl groups that are exposed to water molecules. As a result, the mass loss of the resin is faster. However, this does not mean the internal cross-linked network can be easily destroyed. Furthermore, the cross-linking interactions (chemical or physical) occurring among resin polymers and between resin polymers and wood components, including cellulose, hemi-cellulose and lignin, should not be ignored. Therefore, more branched functional groups means higher interacting intensity. In this sense, even though the crystallinity of the resin occurred in presence of wood, this phenomenon is good for bonding strength. Crystallinity only means strong and ordered interactions occur among linear resin polymers but not between crystalline region and wood. Therefore, drawing a conclusion that low-molar ratio benefits the water resistance is not safe.

**Figure 7:**
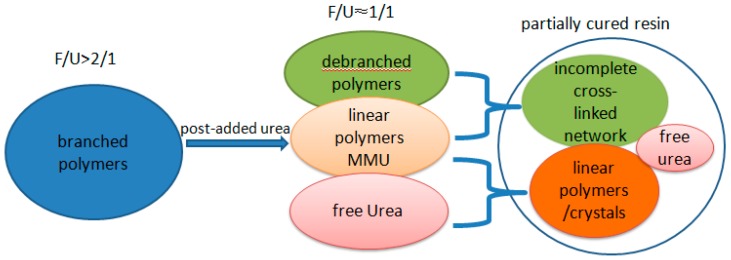
The postulated cure pattern of low-molar ratio urea-formaldehyde (UF) resin.

According the data listed in Table 1, the low-molar ratio UF resin appears to be a mixture of partially branched polymers, a large portion of linear polymers, mono-hydroxymethylurea (MMU), and free urea. The cure of this resin could be described by the pattern in Figure 7. In the curing process, further condensation reactions among the branched polymers formed cross-linked network. Condensation between mono-hydroxymethylureas (MMU) or between urea and MMU mainly produced linear polymers, as found in our previous study [20]. Linear polymers or the linear part of a polymer may also participate in cross-linking reactions, but the contribution would be minor. Moreover, a portion of un-reacted urea may remain in the cured resin, which cannot contribute to the bonding strength. Overall, the cured resin would have a loose structure. As a result, water can easily permeate the partially cured resin resulting in the fast hydrolysis of the polymers and the breakdown of the network. Conversely, the penetration of water also weakens the chemical or physical bonding between the resin and wood components by the competitive interaction and swelling effect.

### 3.2. ESI-MS Spectra

**Figure 8:**
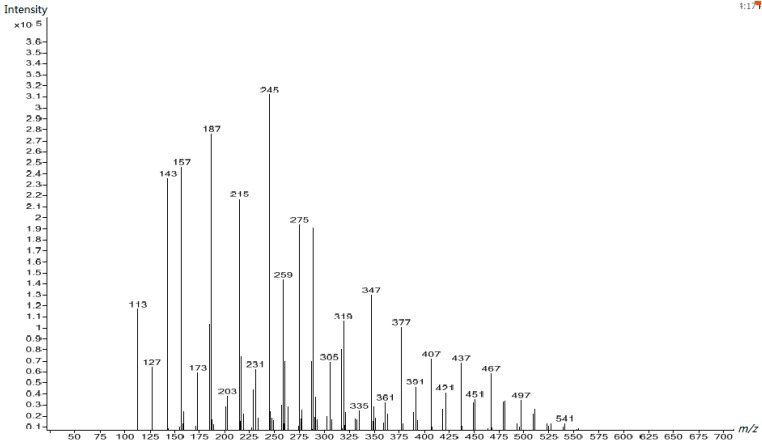
The electrospray ionization mass spectrometry (ESI–MS) spectrum of UF-1.

The ^13^C NMR spectrum for UF1 revealed that the UF reaction under the first alkaline condition produced not only the hydroxymethylureas but also polymers than contain ether linkages. The ESI–MS spectrum of this sample in Figure 8 shows that the molecular masses of the products are distributed in the range of 113 to 541 Da. The assignment for the main peaks are listed in Table 2. The polymers may have isomers and we provide one or two of the most possible species by referring to the ^13^C NMR spectra.

The peaks in the range of 113 to 203 Da correspond to the mono-, di-, and tri- hydroxymethylureas. The peaks at 215–541 Da correspond to the polymers containing ether linkages and uron species with ending urea hydroxymethylated. Apparently, hydroxymethylureas were not the dominant species. Instead, the ether linked prepolymers were the main products.

**Figure 9:**
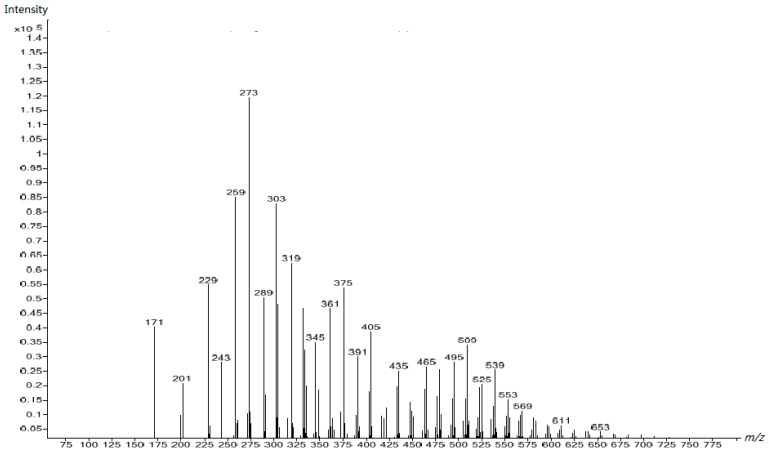
The ESI–MS spectrum of UF-2.

**Figure 10:**
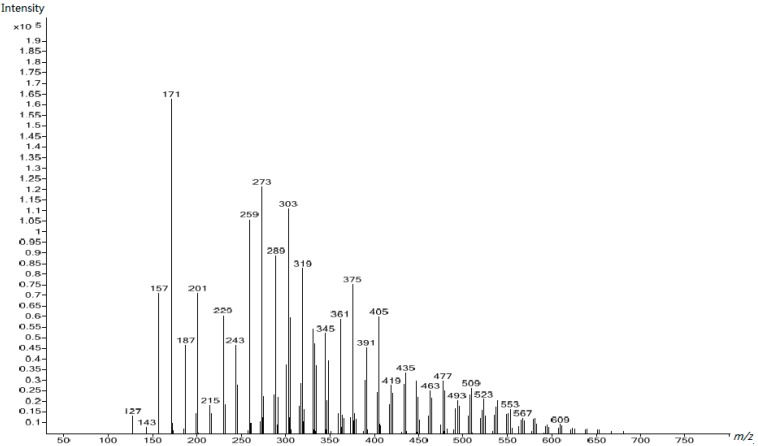
The ESI–MS spectrum of UF-3.

**Figure 11:**
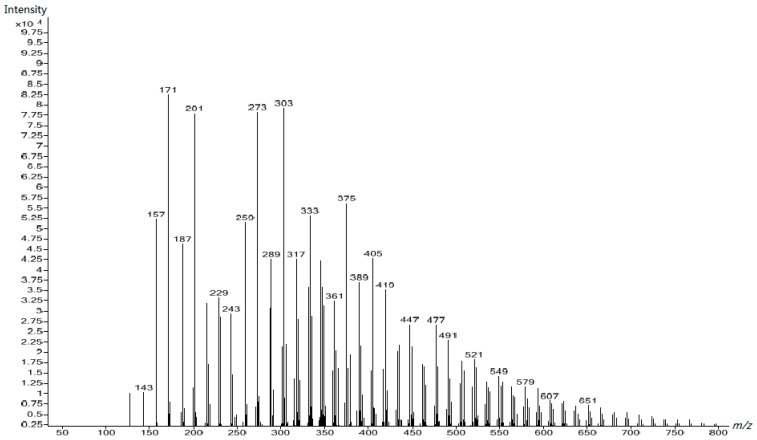
The ESI–MS spectrum of UF-4.

The peaks in the spectra of UF2, UF3, and UF4, shown in Figure 9, Figure 10 and Figure 11 respectively, were obviously different from those in Figure 8, clearly indicating that different species were formed once the reactions occurred in acidic conditions. Besides the increased molecular masses, different chemical structures formed. For comparison, we assigned the peaks of Figure 11 to UF4 in Table 3. The dominant condensed structure is now methylene linkages. This is consistent with the ^13^C NMR results.

**Figure 12:**
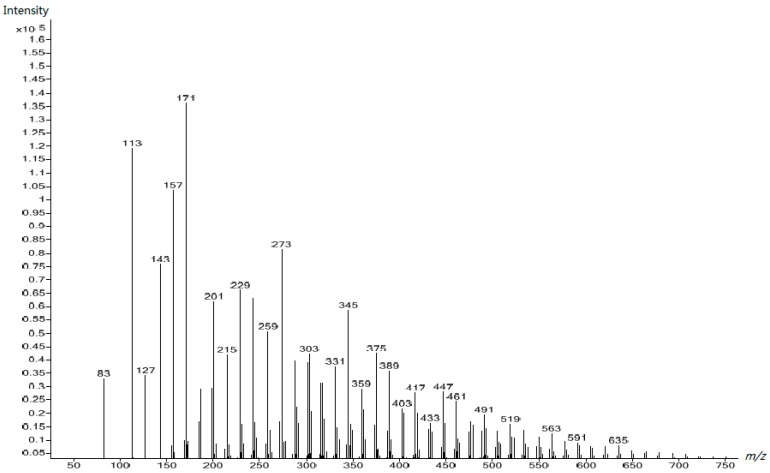
The ESI–MS spectrum of UF-5.

As the ^13^C NMR spectra have shown, the additional urea added at the second alkaline stage caused a significant increase in mono-hydroxymethylurea and a portion of urea remained un-reacted. The ESI-MS spectrum showed that more species with low molecular mass were formed. Figure 12 displays the spectrum of UF5, which was the post-urea treated resin. In contrast to UF4, some distinct changes can be observed. First, a strong peak at 83 Da indicates a large portion of post-added urea was still present and its intensity was even higher than that of the polymers above 400 Da. Second, mono-hydroxymethylurea (113 Da) and di-hydroxymethylurea (143 Da) increased sharply. As discussed above, further condensation may occur between small molecules during the curing process, but the low formaldehyde content determines these condensations mainly form linear polymers with low molecular masses, not cross-linking networks. These polymers, in addition to the monomers, remain soluble in water. This is a negative effect of the post-added urea. Finally, the decrease in the molecular masses in the region of 400–800 Da can also be seen. Specifically, by comparing the peaks in Figure 11 with those in Figure 12, the cleavage of a hydroxymethyl group occurred at the peaks above 300 Da. This is the debranching effect revealed by the ^13^C NMR spectra. Thermodynamically, movement of hydroxymethyl groups from polymers to urea occurs due to the monomeric N-substituted structure being more stable than the N,N-substituted structure. In other words, the primary amino group (–NH_2_) is more reactive than the secondary amino group (–NHR) toward formaldehyde due to the steric hindrance effect.

### 3.3. A Proposed Alternative to Post-Added Urea

**Figure 13:**
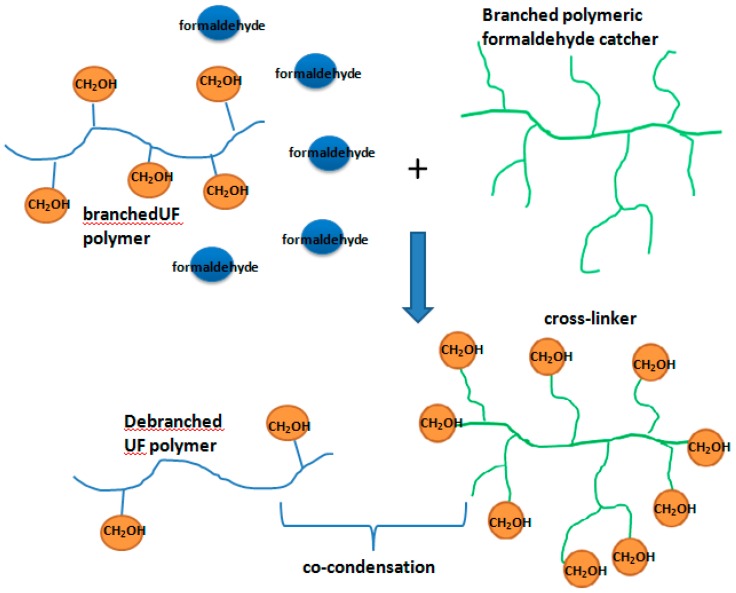
The proposed branched polymeric formaldehyde catcher.

From the ^13^C NMR spectra, after the acidic condensations finished, less than 10% free formaldehyde was present, but almost 100% post-urea appears to be necessary to ensure very low levels of formaldehyde emission due to the reversibility of the hydroxymethylation and condensation reactions. In this sense, the post-added urea acts mainly as a formaldehyde catcher. In addition to urea, other small molecules like melamine and phenol are also frequently used as scavengers. Such scavengers can “catch” both free formaldehyde and hydroxymethyl groups if the catcher is a urea-type catcher or has higher reactivity than urea. That is to say, use of any catcher would cause branched UF polymers to be more linear. Therefore, the use of a catcher should never be “the more the better”. However, it will be much better if the catcher itself has a highly branched structure and co-condensations can occur between the hydroxymethylated catcher and the UF components. Namely, the branched structure of the catcher allows it act as a cross-linker that can offset the debranching effect on the UF polymers. The speculated use of this catcher is demonstrated in Figure 13. The highly branched catcher can be used as an alternative to the post-added urea and the cross-linking function may contribute to the bonding strength of the resin, avoiding the reduction of the resin performance. Thus, this type of catcher is worthy of considerable attention.

Some hyper-branched polymers, with many ending amino groups and good solubility in water, like hyper-branched poly(amidoamines) (PAMAM) generally synthesized through the reaction between methyl acrylate and ethylenediamine, may be potential good formaldehyde catchers and cross-linkers. A hyper-branched polymer was used to modify melamine–urea-formaldehyde resin in an earlier study [25]. The results showed that the PAMAM did not contribute to the dry internal bonding strength of particleboard, and improvement of the wet bonding strength was also not significant. The reactive amino group (–CH_2_–CH_2_–NH_2_) enables it to be a good formaldehyde catcher, but according to our later study on the mechanism of UF condensation reactions [19], the aliphatic nature of the amino group create a challenge for the hydroxymethylated groups (–CH_2_–CH_2_–NHCH_2_OH) to co-condense with UF components. Therefore, three basic requirements, branched structure, good water solubility, and urea-like reactivity, must be satisfied. Guided by these requirements, creating a suitable polymer that acts as both a formaldehyde catcher and cross-linker may be possible.

## 4. Conclusions

The structural changes during a three-step synthesis of a low-molar ratio UF resin were tracked by quantitative ^13^C NMR analysis and ESI–MS. We found that the condensations that produced polymers linked by ether bonds were notable in addition to the hydroxymethylation reactions at the first alkaline stage with F/U = 2:1. A simple word “hydroxymethylation” in the classic theory does not appear to be appropriate to describe the alkaline UF reactions.

After the reactions were brought to the acidic stage, methylene linkages began to form, and became dominant at the end of this stage. The considerable formation of branched methylene linkages, that had the highest content among all the condensed structures, was the key feature of this stage. Notably, the urons content remained the same during this stage, indicating it is high stability.

Changes were observed in the chemical structures and molecular masses of the resin components after the F/U molar ration was lowered to 1.2 by adding post-urea at the final alkaline stage. Specifically, the hydroxymethyl groups on the polymers were cleaved, resulting in a significant decrease in the branching degree of the polymers. The performance degradation of the UF resin was attributed to the debranching effect and the production of components with low molecular masses. The curing pattern of the low-molar ratio UF resin was postulated based on the observations. A possible solution to the debranching effect is to replace the post-added urea with some kind of polymer with a highly branched structure and urea-like reactivity. This polymer could then act as both a formaldehyde catcher and a cross-linker.

## Figures and Tables

**Table 1:** Relative content of the methylenic and carbonyl carbons (%).

Structure	Chemical Shift (ppm)	UF-1	UF-2	UF-3	UF-4	UF-5
–NH–CH_2_–NH–(I)	46–47	-	4.89	7.57	10.82	15.16
–NH–CH_2_–N=(II)	53–54	-	3.48	8.03	23.76	16.10
=N–CH_2_–N=(III)	60–61	-	-	-	0.68	-
	Total	-	8.37	15.60	35.26	31.26
–NH–CH_2_OCH_2_NH–(I)	68–69	18.26	20.31	18.61	10.74	10.69
–NH–CH_2_OCH_2_N=(II)/uron	75–76	2.38	2.42	3.05	1.40	2.40
=N–CH_2_OCH_2_N=(III)/uron	78–80	6.71	7.32	8.13	9.98	4.14
	Total	27.35	30.05	29.79	22.12	17.23
–NH–CH_2_OH(I)	64–65	55.63	39.81	31.32	16.37	46.59
–NH(–CH_2_)–CH_2_OH(II)	70–71	9.04	9.72	11.03	13.94	1.82
	Total	64.67	49.53	42.35	30.31	48.41
HO–CH_2_–OH	83–84	1.13	1.57	1.93	3.99	0.24
HOCH_2_–O–CH_2_–OCH_2_OH	86–87	1.84	1.54	1.99	3.00	0.30
HOCH_2_–O–CH_2_–OCH_2_OH	90–91	2.43	1.26	1.53	2.20	0.21
H(CH_2_O)*_n_*OCH_2_OCH_3_	94–95	0.48	0.39	0.31	-	-
	Total	5.88	4.76	5.76	9.19	0.75
–NH–CH_2_–O–CH_3_	72–73	2.11	7.27	6.51	3.12	2.35
NH_2_–CO–NH_2_	162–163	4.29	1.31	0.48	-	19.94
NH_2_–CO–NH–	160–162	33.74	29.85	15.52	4.42	38.52
–NH–CO–N–/–NH–CO–N=	159–160	54.55	61.38	76.46	88.22	38.47
Uron	153–158	7.42	7.46	7.54	7.36	3.07

**Table 2:** Assignment for the electrospray ionization mass spectrometry (ESI–MS) peaks of UF reaction products at first alkaline stage (Sample 1).

*m*/*z*	Structure	*m*/*z*	Structure
113	U–CH_2_OH (Na^+^)	275	HOCH_2_–U–CH_2_O–CH_2_–U–(CH_2_OH)_2_ (Na^+^)
127	U–CH_2_OH (H^+^)…2H_2_O	289	Uron–CH_2_–O–CH_2_–U–CH_2_OH (K^+^)
143	HOCH_2_–U–CH_2_OH (Na^+^)	305	(HOCH_2_)_2_–U–CH_2_O–CH_2_–U–(CH_2_OH)_2_ (Na^+^)
157	CH_2_OH–U–CH_2_OH (H^+^)…2H_2_O	319	Uron-CH_2_–O–CH_2_–U–(CH_2_OH)_2_ (K^+^)
173	HOCH_2_–U–(CH_2_OH)_2_ (Na^+^)	335	(HOCH_2_)_2_–U–CH_2_O–CH_2_–U–(CH_2_OH)–CH_2_–O–CH_2_OH (Na^+^)
187	CH_2_OH–U–(CH_2_OH)_2_ (H^+^)…2H_2_O	347	(HOCH_2_)_2_–U–CH_2_O–CH_2_–U–CH_2_–O–CH_2_–U (Na^+^)
203	HOCH_2_–U–CH_2_OH (Na^+^)…U	361	Uron–CH_2_–O–CH_2_–U–CH_2_–O–CH_2_–U (K^+^)
215	HOCH_2_–U–CH_2_O–CH_2_–U (Na^+^)	377	(HOCH_2_)_2_–U–CH_2_O–CH_2_–U–CH_2_–O–CH_2_–U–CH_2_OH (Na^+^)
231	HOCH_2_–U–CH_2_O–CH_2_–U (K^+^)	391	Uron–CH_2_–O–CH_2_–U–CH_2_–O–CH_2_–U–CH_2_OH (K^+^)
245	HOCH_2_–U–CH_2_O-CH_2_–U–CH_2_OH (Na^+)^	407	(HOCH_2_)_2_–U–CH_2_O–CH_2_–U–CH_2_–O–CH_2_–U–(CH_2_OH)_2_ (Na^+^)
259	Uron–CH_2_–O–CH_2_–U (K^+^)	421	Uron–CH_2_–O–CH_2_–U–CH_2_–O–CH_2_–U–(CH_2_OH)_2_ (K^+^)

**Table 3:** Assignment for the ESI–MS peaks of the UF reaction products at the acidic stage (Sample 4).

*m*/*z*	Structure	*m*/*z*	Structure
143	CH_2_OH–U–CH_2_OH (Na^+^)	361	HOCH_2_–U–CH_2_–U–CH_2_–U–(CH_2_OH)–CH_2_–U (H^+^)…H_2_O
157	CH_2_OH–U–CH_2_OH (H^+^)‥.2H_2_O	375	Uron–CH_2_OH+CH_2_OH–U–(CH_2_OH)_2_+U–CH_2_OH\HOCH_2_–U–CH_2_–U–CH_2_–U–(CH_2_OH)–CH_2_–U (K^+^)
171	Uron–CH_2_OH (K^+^) U–CH_2_–U (K^+^)	389	HOCH_2_–U–CH_2_–U–CH_2_–U–(CH_2_OH)–CH_2_–U–CH_2_OH (Na^+^)
187	CH_2_OH–U–(CH_2_OH)_2_ (H^+^)…2H_2_O	405	HOCH_2_–U–CH_2_–U–CH_2_–U–(CH_2_OH)–CH_2_–U–CH_2_OH (K^+^)
201	Uron–(CH_2_OH)_2_\U–CH_2_–U–CH_2_OH (K^+^)	419	HOCH_2_–U–CH_2_–U–CH_2_–U–(CH_2_OH)–CH_2_–U–(CH_2_OH)_2_ (Na^+^)
229	HOCH_2_–U–(CH_2_OH)–CH_2_–U (H+)…2H_2_O	447	HOCH_2_–U–CH_2_–U–CH_2_–U–(CH_2_OH)–CH_2_–U–CH_2_–U (K^+^)
243	HOCH_2_–Uron–CH_2_–U\U–CH_2_–U–CH_2_–U (K^+^)	477	HOCH_2_–U–CH_2_–U–CH_2_–U–(CH_2_OH)–CH_2_–U–CH_2_–U–CH_2_OH (K^+^)
273	HOCH_2_–Uron–CH_2_–U–CH_2_OH\HOCH_2_–U–CH_2_–U–CH_2_–U (K^+^)	491	(HOCH_2_)_2_–U–CH_2_–U–CH_2_–U–(CH_2_OH)–CH_2_–U–CH_2_–U–CH_2_OH (Na^+^)
303	HOCH_2_–Uron–CH_2_U–(CH_2_OH)_2_\HOCH_2_–U–CH_2_–U–CH_2_–U–CH_2_OH (K^+^)	521	(HOCH_2_)_2_–U–CH_2_–U–CH_2_–U–(CH_2_OH)–CH_2_–U–CH_2_–U–(CH_2_OH)_2_ (Na^+^)
317	HOCH_2_–U–CH_2_–O–CH_2_–U–CH_2_OH\CH_2_OH–U–CH_2_–U–(CH_2_OH)_2_ (Na^+^)…2H_2_O	549	HOCH_2_–U–CH_2_–U–CH_2_–U–CH_2_–U–(CH_2_OH)–CH_2_–U–CH_2_–U–CH_2_OH (K^+^)
333	HOCH_2_–U–CH_2_–U–CH_2_–U–(CH_2_OH)_2_ (K^+^)	579	(HOCH_2_)_2_–U–CH_2_–U–CH_2_–U–CH_2_–U–(CH_2_OH)–CH_2_–U–CH_2_–U–CH_2_OH (K^+^)
